# Global Distribution of Three Parasitoids of *Drosophila suzukii* (Diptera, Drosophilidae): Present and Future Climate Change Scenarios

**DOI:** 10.3390/insects17010012

**Published:** 2025-12-21

**Authors:** Lenon Morales Abeijon, Jesús Hernando Gómez-Llano, Sergio Marcelo Ovruski, Flávio Roberto Mello Garcia

**Affiliations:** 1 Programa de Pós-Graduação em Fitossanidade, Universidade Federal de Pelotas, Pelotas 96000-010, RS, Brazil; lenonm.faem@ufpel.edu.br (L.M.A.); jhgomezl@unal.edu.co (J.H.G.-L.); 2 Pilot Plant of Industrial Microbiological and Biotechnology Processes (PROIMI-CONICET), Biological Control Department, Avda. Belgrano and Pje. Caseros, San Miguel de Tucumán T4001MVB, Argentina; sovruski@conicet.gov.ar; 3 Departamento de Ecologia, Zoologia e Genética, Instituto de Biologia, Universidade Federal de Pelotas, Capão do Leão 96160-000, RS, Brazil

**Keywords:** biocontrol agents, climate change, environmental variables, natural enemies, parasitoids, random forest, species distribution modeling, spotted wing drosophila

## Abstract

In this study, we investigated the current and future potential distribution of three parasitoid species of *Drosophila suzukii*, which represent promising candidates for the biological control of this pest: *Leptopilina japonica* (Hymenoptera, Figitidae), *Pachycrepoideus vindemmiae* (Hymenoptera, Pteromalidae), and *Trichopria drosophilae* (Hymenoptera, Diapriidae). To this end, we employed Ecological Niche Modeling using the Random Forest algorithm and climatic data from WorldClim v. 2.1 under climate change scenarios (SSP2-4.5 and SSP5-8.5), analyzing the spatial overlap between the pest and its natural enemies. The results indicate that the parasitoids exhibit distinct geographic distributions, although most species show higher suitability for temperate regions of the Northern Hemisphere. Species such as *T. drosophilae* and *L. japonica* stand out for their broad distribution and high overlap with the pest, whereas *P. vindemmiae* and display more restrictive climatic ranges and lower control efficiency. With ongoing climate change, all parasitoids tend to migrate toward higher latitudes, with significant range contractions in tropical regions. Thus, our results demonstrate the usefulness of Ecological Niche Modeling in the selection of biological control agents by considering host-specific preferences and environmental requirements in the development of management strategies adapted to future scenarios.

## Simple Summary

The spotted-wing drosophila (*Drosophila suzukii*) is an invasive agricultural pest that causes significant damage to crops in various parts of the world by infesting fruits that are still ripening. Controlling this species has been a major challenge, particularly due to the limitations and environmental impacts associated with excessive pesticide use. As an alternative, several biological control methods have been proposed. These methods rely on the release of the pest’s natural enemies, such as parasitoids, which lay their eggs inside the fly, preventing its development. In this study, we employed Ecological Niche Modeling techniques to analyze the present and future potential distribution of three *D. suzukii* parasitoid species, aiming to contribute to the selection of the most suitable candidates for the pest’s biological control. The projections obtained indicate that some of these natural enemies are capable of surviving in many regions of the world, while others have more restrictive environmental requirements. With the expected temperature increases in the coming decades, these insects may migrate to more northern areas, ceasing to occupy tropical regions. The results help identify which species are best suited to control the pest in different locations in the short and medium term. This type of research is essential for selecting the most effective biological control agents, contributing to crop protection in a more natural and sustainable way, and ensuring safer food with lower environmental impact.

## 1. Introduction

In recent decades, there has been a significant increase in agricultural production worldwide [1]. However, this progress has often been hindered by the presence of a variety of insect pests that cause reductions in crop productivity [2,3]. Global warming and increasing economic globalization have accelerated the proliferation of new invasion routes for pests [4], resulting in additional challenges for agriculture. Given the serious threat that pests pose to global systems [5], several strategies have been developed for their management, each with its own advantages and disadvantages [6].

The spotted-wing drosophila (SWD), *Drosophila suzukii* (Matsumura, 1931) (Diptera: Drosophilidae), is a harmful species to agricultural crops that has become a global pest in the last decade [7]. Native to Southeast Asia, this species is known for its ability to invade new territories [7,8,9,10,11], driven by its high potential for geographic dispersal [12] and its tolerance to a wide range of climatic conditions [13]. Moreover, it is capable of attacking a wide variety of intact ripe fruits [14], damaging them by piercing the epicarp for oviposition, leading to highly significant economic losses [15,16].

Currently, the management of *D. suzukii* relies on the application of synthetic insecticides such as pyrethroids, organophosphates, spinosyns, and neonicotinoids [17,18]. However, frequent application of these conventional insecticides can cause negative impacts on the environment and human health [19,20]. Given the growing interest in organic control methods that represent lower ecological damage while remaining effective in pest management, it is necessary to develop and adopt safer and more ecologically sustainable strategies [21,22,23]. These alternatives should combine food safety properties, lower environmental impact, economic feasibility, and long-term solutions [24].

Among the methods employed in *D. suzukii* management, classical biological control stands out. It consists of the release of natural enemies from the pest’s region of origin (Asia) as a means to reduce population growth in invaded areas [4,25,26]. This approach aims to increase the effectiveness of crop management strategies while reducing the negative side effects of chemical control [27]. Through the exploration of various natural enemies of *D. suzukii* in its native range, parasitoids from the genera *Asobara* (Braconidae), *Ganaspis*, *Leptopilina* (Figitidae), *Trichopria* (Diapriidae), and *Pachycrepoideus* (Pteromalidae) have been widely studied as potential biological control agents of *D. suzukii* [25,26,28,29,30,31,32].

In this context, some researchers discourage the use of *Asobara japonica* Belokobylskij, 1998 in *D. suzukii* control programs [4,25,33], due to its wide host range [34,35]. Furthermore, in a coexistence scenario among the three species, *Leptopilina japonica* Förster, 1869 is the only one capable of surpassing the others due to its relatively faster egg hatching potential [36]. In addition to these parasitoids, several studies have highlighted the ability of *Trichopria drosophilae* (Perkins, 1910) and *Pachycrepoideus vindemmiae* (Rondani, 1875) to reduce *D. suzukii* populations under both laboratory and field conditions [37,38,39,40,41].

Field studies by Rossi-Stacconi et al. [37,42] show that *T. drosophilae* is effective even in unmanaged areas and across a range of elevations. Zhu et al. [43] corroborated this by demonstrating strong reproductive performance under varying conditions, with high parasitism rates, a short mean generation time (21–29 days), and a significant proportion of female offspring. Nevertheless, laboratory studies such as Wang et al. [39] indicate that, despite being a specialist on SWD [40] and its reproductive capacity, *T. drosophilae* can be outcompeted by other parasitoids, such as *P. vindemmiae*, in competitive interactions.

Ecological Niche Modeling (ENM) has been recognized as an excellent tool for identifying the potential distribution of species for various purposes [44,45,46,47,48,49,50], including elucidating dispersal patterns and establishing suitable strategies for integrated pest management [8,50]. By enabling the selection of appropriate sites for the release of natural enemies based on their environmental requirements, the use of these tools can increase the success of biological control agents [51]. In fact, when applied to the context of pests and parasitoids, these tools allow for the identification and comparison of the relative suitability of pests and parasitoids across different habitats, thus guiding and enhancing the development of more effective biological control programs [52,53,54].

Furthermore, when focused on the effects of climate change on insects, these models are widely used to predict species distribution patterns [55], employing different statistical methods such as CLIMEX and Random Forest (RF) [56,57]. These projections allow the assessment of areas with climatic conditions favorable for biological control and the release of parasitoids [57].

However, although CLIMEX (version 3) is widely known and used, it is not a free software program, which limits its use for logistical reasons [57,58]. In contrast, RF operates by forming an ensemble of unpruned classification or regression trees built using bootstrap samples of training data and random selection of features during tree induction. In terms of performance, RF is one of the most accurate tree-based classification and regression models. The predictors generated by RF trees are combined such that each depends on the values of independently sampled random vectors, assuming a similar distribution for each tree in the forest. The aggregation (mean) of predictions across the ensemble forms the basis of the final prediction. Individual observations from each tree are used to estimate model error, and variable importance and decision-tree predictions are averaged. Unlike CLIMEX, RF is a free software tool, making it a viable option [58,59].

Therefore, this study aims to model the current and future potential distribution of three larval and pupal parasitoid species of SWD: *L. japonica*, *P. vindemmiae*, and *T. drosophilae*, seeking to infer the degree of overlap between each parasitoid and its host species. These models will allow the prediction of areas with potential suitability for these species, providing valuable data for future pest management strategies.

## 2. Materials and Methods

All procedures related to data compilation and processing, model development, and map creation were conducted in the R environment, version 4.3.2 [60].

### 2.1. Occurrence Data of Spotted Wing Drosophila Parasitoids

Occurrence records of the species *L. japonica*, *P. vindemmiae*, and *T. drosophilae* were collected from five databases: the Global Biodiversity Information Facility (GBIF, Copenhagen, Denmark; https://www.gbif.org/, accessed on 15 December 2024), iNaturalist (iNat, California Academy of Sciences, San Francisco, CA, USA; https://www.inaturalist.org/, accessed on 15 December 2024), VertNet (VertNet, University of Florida, Gainesville, FL, USA; http://www.vertnet.org/, accessed on 15 December 2024), Berkeley Ecoinformatics Engine (Ecoengine, University of California, Berkeley, CA, USA; https://bnhm.berkeley.edu/informatics/ecoengine/, accessed on 15 December 2024), and Integrated Digitized Biocollections (iDigBio, University of Florida; https://www.idigbio.org/, accessed on 15 December 2024). Additional searches were conducted in scientific articles (Supplementary Material—File S1) [4,26,33,43,61,62,63,64,65,66,67,68,69,70,71,72,73,74,75]. The data were retrieved using the “occ” function from the “spocc” package v.1.2.2 [76] and subsequently refined using the “clean_coordinates” function from the “CoordinateCleaner” package v.3.0.1.

Due to the lack of true absence data, ‘pseudo-absences’ were generated within the pest’s occurrence area for each parasitoid species studied, using the ‘randomPoints’ method from the ‘dismo’ package v.1.3-14 in R [77]. The number of pseudo-absences was standardized as twice the number of compiled occurrence records for each species [78].

### 2.2. Climate Layers

The climatic data used for current and future predictions were obtained from WorldClim v. 2.1 (Global Climate Data; [79]) at a spatial resolution of 2.5 arc-minutes (approximately 4.5 km). These data comprise 19 bioclimatic variables (files ‘*.raster’) encompassing temperature and precipitation variables (Table 1).

For current predictions, we used layers covering climatic data recorded between 1970 and 2000. For future projections, we used three global climate models (GCMs) from the Coupled Model Intercomparison Project Phase 6 (CMIP6) [available at WorldClim (https://www.worldclim.org/data/cmip6/cmip6climate.html, accessed on 29 May 2022); [80]]: ACCESS-ESM1-5, HadGEM3-GC31-MM, and MIROC6. In each case, two Shared Socioeconomic Pathways (SSPs) were considered—SSP2-4.5 and SSP5-8.5—which represent moderate and pessimistic scenarios, respectively, with increasing greenhouse gas emissions under a fossil fuel–dependent economy [81,82]. Predictions were made for two 20-year time intervals: 2021–2040 and 2041–2060.

To mitigate multicollinearity in our models, we extracted bioclimatic variable values from the occurrence data of each species. Subsequently, we performed a collinearity test among the variables using the ‘vifstep’ function from the ‘usdm’ package [v1.1-18; [80]]. This step aimed to identify and remove correlated predictor variables in the statistical model [83], which could potentially inflate standard errors and confidence intervals, thereby affecting the determination of the significance of variables in relation to the dependent variable (occurrences) [84]. Only variables with variance inflation factor (VIF) values < 10 were selected for further analyses [85] (Table 1). The VIF value is given by 1/(1 − ri^2^), where ri^2^ represents the coefficient of determination of the predictor variable in relation to the other variables [86].

### 2.3. Current and Future Potential Distribution Modeling

For the ENM, we used the Random Forest algorithm as implemented in the ‘sdm’ package [87]. Random Forests involves numerous parameters that influence both the structure of individual trees and the overall composition of the forest, including their randomization properties. Parameters such as node size, the total number of trees (ntree), and the number of randomly selected candidate variables (mtry) were tuned using the ‘caret’ package v.7.0-1 [88,89] to identify the optimal configuration for a given dataset and prediction task. This procedure resulted in a ntree of 2500 and mtry values of 3 and 25. In each case, 70% of the occurrence data were used for training and 30% for testing.

To delineate suitable areas for each species across different time periods and climate scenarios, we applied a threshold based on the Minimum Training Presence (MTP). This threshold identifies the lowest predicted suitability value associated with an occurrence point, essentially assuming that the least suitable habitat in which the species is found represents the minimum suitability value for that species [90]. This approach allowed us to classify the climatic suitability maps, with values ranging from the MTP to 1 displayed along a green-to-red gradient. Green areas (values close to 1) were identified as highly suitable, yellow areas (values around 0.5) as moderately suitable, areas near the species-specific threshold as having low suitability, and areas below the threshold as unsuitable. Subsequently, binary maps were generated based on each species’ MTP value, classifying areas as either suitable or unsuitable.

### 2.4. Model Evaluation

To evaluate model performance, we employed Receiver Operating Characteristic (ROC) curve analysis, focusing on the Area Under the Curve (AUC) [91] and the True Skill Statistic (TSS) [92]. The AUC value serves as a standard measure for assessing the accuracy of potential distribution models; values below 0.7 are considered poor, between 0.7 and 0.9 moderate, and greater than 0.9 good [93]. TSS is calculated by generating a confusion matrix composed of the number of correct and incorrect predictions for presence and absence areas, applying a classification threshold [91] defined by the sum of sensitivity and specificity [94]. Accordingly, TSS values range from −1 to 1, with TSS ≥ 0.4 considered reasonable, TSS ≥ 0.5 reliable, and TSS ≥ 0.8 indicating excellent performance [95]. For each species, we determined the relative importance of the selected bioclimatic variables based on the AUC and TSS metrics.

### 2.5. Estimation of the Potential Overlap Between the Parasitoids and SWD

The potential overlap between SWD and the parasitoid species was estimated from continuous suitability maps using the threshold determined for each species. These maps were converted into binary maps, with presence (1) and absence (0) values. For the parasitoids, we used the models generated in this study; for *D. suzukii*, we used the presence areas modeled by Abeijon et al. [96], who applied the same methodological approach.

To avoid ambiguities in the overlap analysis, the binary maps were reclassified: pixels showing SWD presence were assigned the value 10, while those indicating parasitoid presence were assigned the value 20; absence values were kept as 0 (zero). The reclassified layers were then summed, pixel by pixel, resulting in four interpretable categories: 0 (absence of both species), 10 (presence of SWD only), 20 (presence of the parasitoid only), and 30 (presence of both). This approach allows for the identification of pest risk areas, potential control areas of the parasitoid species, and regions with no mutual occurrence.

## 3. Results

**Figure 1:**
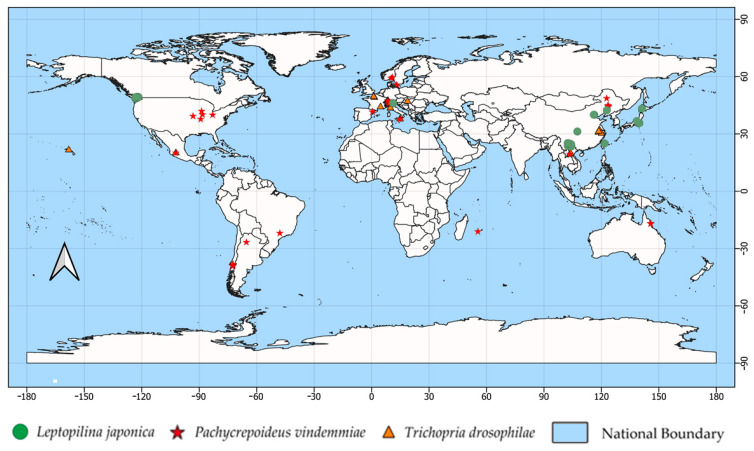
Current distribution of *Drosophila suzukii* parasitoids. Known occurrence points used to construct the predictive models are represented in green (*L. japonica*), red (*P. vindemmiae*), and orange (*T. drosophilae*).

The search for *D. suzukii* parasitoid records across the five databases and published articles resulted in 151 geographic coordinates, distributed as follows: 41 for *L. japonica*, 63 for *P. vindemmiae*, and 47 for *T. drosophilae* (Figure 1; Table 2; Supplementary File S1). Regarding the bioclimatic variables with the greatest contribution in each case, BIO13 contributed the highest percentage to the *L. japonica* model (32.0%); BIO2 to *T. drosophilae* (24.6%); and BIO19 to *P. vindemmiae* (22.7%) (Table 3).

### 3.1. Current Potential Distribution of D. suzukii Parasitoids

Our models showed excellent performance, with AUC values ranging from 0.988 ± 0.020 (for *L. japonica)* to 0.981 ± 0.026 (for *P. vindemmiae*), and TSS values ranging from 0.550 for *T. drosophilae* to 0.530 for *P. vindemmiae* (Table 2).

**Figure 2:**
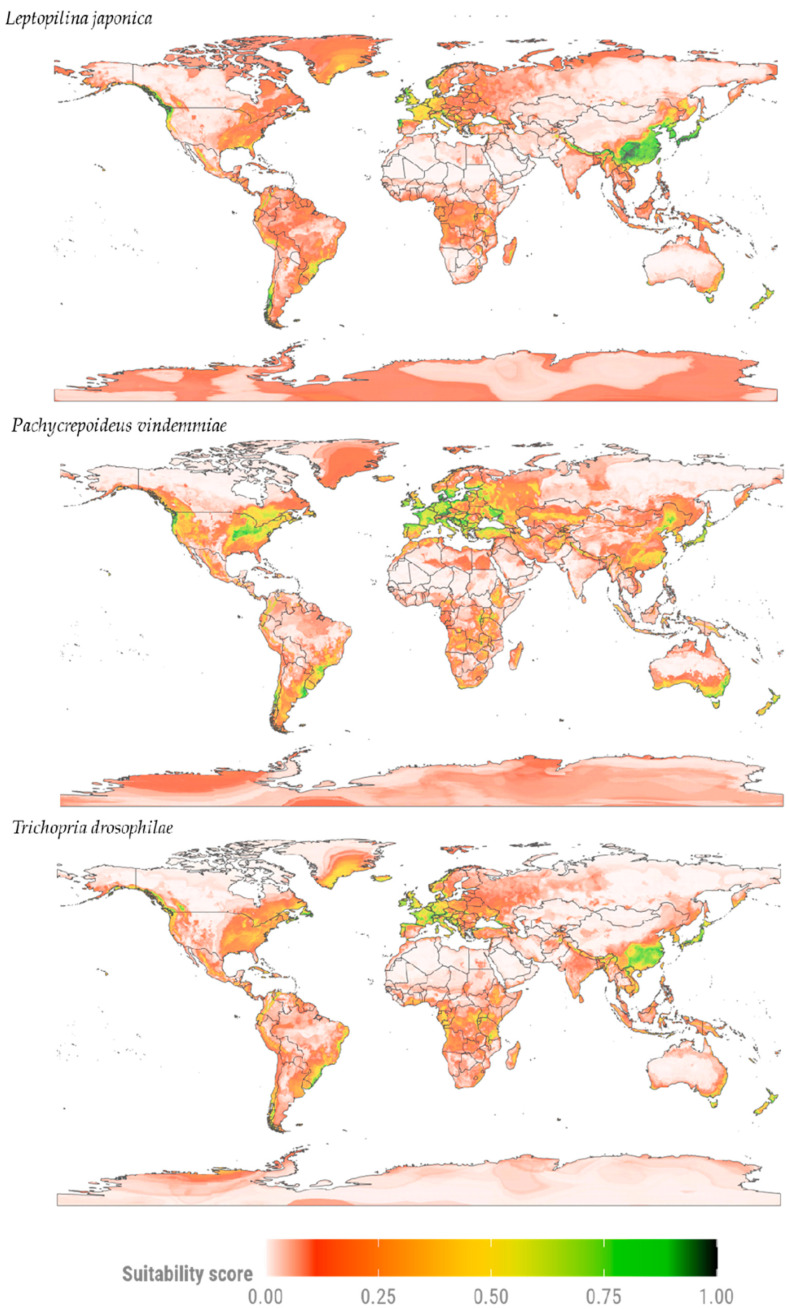
Potential distribution maps of *D. suzukii* parasitoids modeled under current conditions for *L. japonica*, *P. vindemmiae*, and *T. drosophilae*. Suitability values vary according to a red-to-green color scale, with red indicating low suitability and green indicating high suitability.

Our distribution models indicate that the parasitoid species exhibit distinct distributions, with the highest climatic suitability values concentrated in North America, Europe, and Asia—mainly along the Eastern and Northwestern Coasts of the United States and in Canada (Figure 2). In Europe, areas with high suitability are predicted in Central and Eastern regions, while in Asia—the native continent of SWD—high suitability values occur in some areas near China and Japan. Some species also show potential to occupy parts of South America and Oceania. In South America, areas with lower suitability are found in some regions of Brazil and in isolated areas of the Andes.

The parasitoids *L. japonica* and *P. vindemmiae* show a broader potential distribution compared to *T. drosophilae*, with the highest environmental suitability values distributed between Europe and Asia. *Pachycrepoideus vindemmiae* stands out for its high suitability in areas of Eastern United States, Southeastern Australia, and Southern South America. Conversely, *L. japonica* is characterized by high suitability in regions with humid temperate, humid subtropical, and monsoonal climates, primarily in East Asia. Finally, areas with high climatic suitability values for *T. drosophilae* are distributed across different parts of the globe, especially in Eastern North America, Western Europe, Far East Asia, and in countries such as Brazil (South America) and Tanzania and Mozambique (Africa).

### 3.2. Potential Distribution of SWD Parasitoids Under Two Climate Change Scenarios

**Figure 3:**
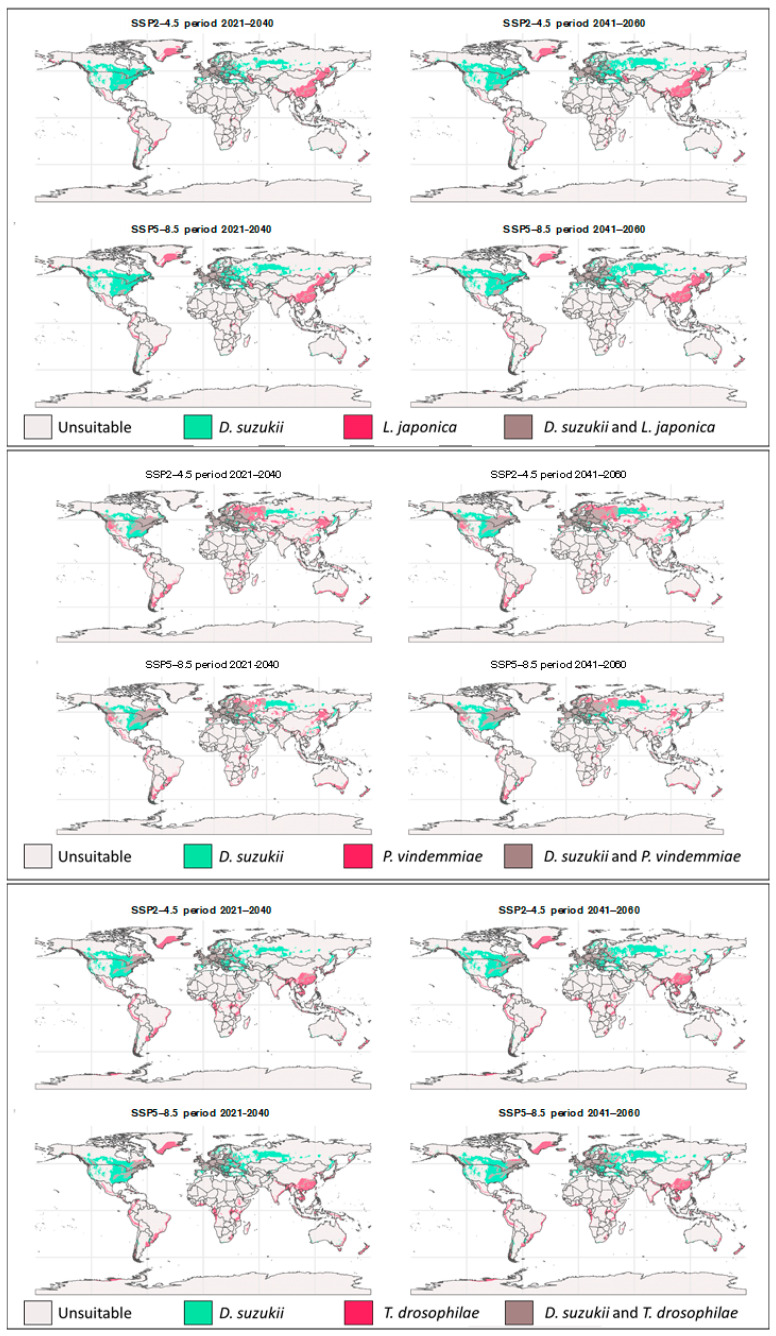
Future suitability maps for the parasitoid species *L. japonica*, *P. vindemmiae*, and *T. drosophilae*, and their overlap with *D. suzukii* under climate change scenarios SSP2-4.5 and SSP5-8.5 across two intervals (2021–2040 and 2041–2060).

The projections of climatic suitability for the parasitoids under future scenarios show significant changes for the periods 2021–2040 and 2041–2060, in both SSP2-4.5 (moderate mitigation) and SSP5-8.5 (high emission) scenarios allowing comparison through Figure 3 and the percentual suitable area shown in Tables S1–S3 (Supplementary File S2).

#### 3.2.1. Moderate Mitigation Scenario (SSP2-4.5)

Under the moderate mitigation scenario, a general trend of expansion of climatically suitable areas toward mid and high latitudes was observed, with a moderate reduction in tropical regions. For *L. japonica*, the future models built under the moderate scenario for 2021–2040 project the maintenance of high climatic suitability values across East Asia (China and Japan), Central Europe, and the Eastern United States. Nonetheless, there is a slight expansion of these areas toward higher northern latitudes, such as southern Canada, Northern Europe, and central China. Tropical regions continue to show low climatic suitability.

*Pachycrepoideus vindemmiae* showed an expansion of climatically suitable areas in Scandinavia and Northern Europe, as well as in Northern United States and Southern Canada, while other regions became less suitable for parasitoid establishment. The species also shows a reduction in suitable areas in tropical regions of South America, Africa, and Southeast Asia up to 2040. This pattern appears to intensify between 2041–2060, when Southern South America emerges as the main area of suitability in Latin America, encompassing the Argentine Pampas, Southeastern Brazil, and the austral zone of Chile.

Finally, future climatic suitability areas for *T. drosophilae* are distributed mainly across temperate regions, indicating the potential establishment of the species in Central Europe, the eastern United States, and Eastern China.

#### 3.2.2. Pessimistic Mitigation Scenario (SSP5-8.5)

Under the high carbon emission scenario, a more pronounced shift in climatically suitable areas toward mid and high latitudes was observed, with a significant reduction in tropical and subtropical regions. *Leptopilina japonica*, during the 2041–2060 period, climatic suitability intensifies in mid- and high-latitude areas, particularly in Northern Europe, western Russia, and central and boreal regions of Asia (Figure 3). In North America, Canada begins to show more continuous suitability patterns, while southern U.S. areas tend to maintain or slightly reduce their suitability. South America retains only small areas of climatic suitability, concentrated in the southern portion of the continent. In Oceania, small suitable patches persist in Southeastern Australia and New Zealand. Tropical regions continue to show low suitability values, suggesting climatic limitation.

*Pachycrepoideus vindemmiae* exhibits one of the clearest patterns of potential expansion toward high-latitude regions. Between 2021–2040 and especially 2041–2060, areas of climatic suitability expand across Northern United States, Canada, Northern Russia, and Scandinavia, forming wide, continuous bands of high suitability. In contrast, a strong decline in suitability is observed across tropical and subtropical regions, including South America, Africa, Southern Asia, and Oceania. In Latin America, only Southern Brazil, Northern Argentina, and the austral zone of Chile remain moderately suitable by the end of the analyzed period.

Finally, *T. drosophilae* follows a similar pattern, maintaining climatically suitable areas in temperate zones such as Central Europe, Northeastern United States, and Eastern China, with a gradual potential colonization of boreal regions in Europe and Asia. Like the other parasitoids, *T. drosophilae* also losses suitable areas across tropical regions, particularly in parts of South America, Africa, and Southeast Asia, where suitability values become very low by 2060.

### 3.3. Overlap of the Current Potential Distribution of SWD and Its Parasitoids

**Figure 4:**
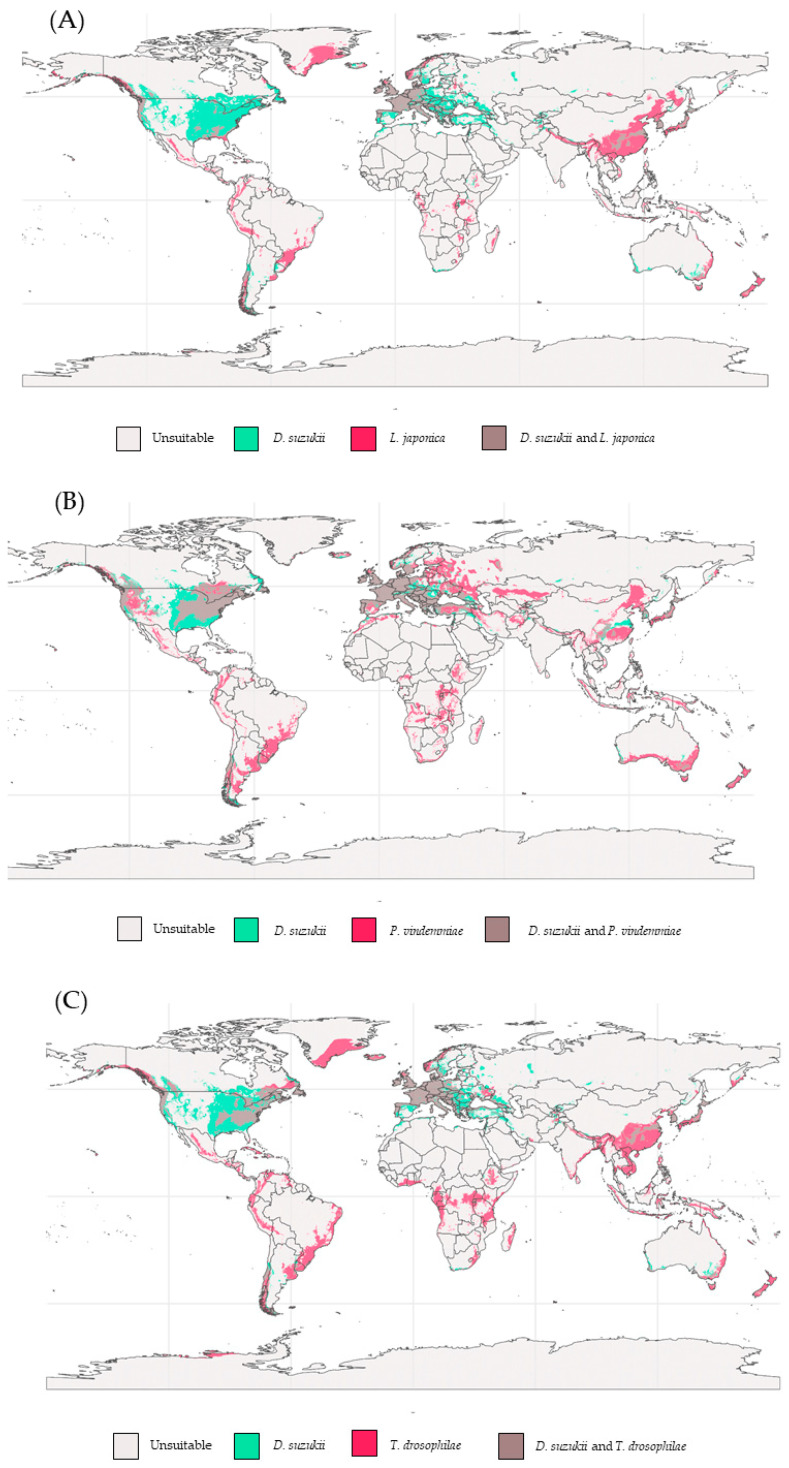
Binary overlap maps of the potential distribution between *D. suzukii* and its parasitoids *L. japonica* (**A**), *P. vindemmiae* (**B**), and *T. drosophilae* (**C**) modeled under current conditions.

The overlap areas between *D. suzukii* and its parasitoids under current conditions vary according to the species analyzed, indicating potential zones for biological control (Figure 4). According to the models, none of the parasitoid species show potential overlap with SWD across most of the African, Oceanian, and South American continents.

*Trichopria drosophilae* shows overlap with *D. suzukii* in North America, particularly in the Eastern and specific Western regions of the United States. In Europe, both species exhibit potential distribution in Central and Southern regions. In Asia, this pattern is repeated in restricted areas of the Far East and Southeast Asia, including South Korea, China, Japan, and India.

The potential overlap between *L. japonica* and *D. suzukii* is more pronounced in temperate regions of the Northern Hemisphere, such as Southeast Asia (China, Japan, and South Korea), Central Europe, and the Western United States. Overlapping potential distribution areas are also observed in Southern Chile, whereas in tropical regions such as Central America and Africa, overlap is limited.

*Pachycrepoideus vindemmiae* exhibits the greatest potential overlap with *D. suzukii* at a global scale, with shared suitability between both species across large regions of North America (Western, Midwestern, and Eastern United States and Southeastern Canada) and Europe. In Asia and South America, the species overlap in specific areas, particularly in Japan and Chile, respectively.

### 3.4. Overlap of the Future Expansion of SWD and Its Promising Parasitoids Under Climate Change Scenarios

The potential overlap areas between each parasitoid species and *D. suzukii* are projected to change in the future across both climate scenarios (SSP2-4.5 and SSP5-8.5) and time intervals (2021–2040 and 2041–2060), showing patterns of both expansion and contraction.

*Leptopilina japonica* shows no expansion or contraction in its potential overlap with the pest during 2021–2040 and 2041–2060 under the moderate scenario (SSP2-4.5). Under a high carbon emission scenario (SSP5-8.5), this parasitoid exhibits a localized expansion of potential overlap areas in Eastern Europe and Western Asia during the 2041–2060 period.

*Pachycrepoideus vindemmiae* stands out for its marked increase in overlap area, covering much of the European continent and the western portion of Asia. Finally, the overlap areas between the pest and *T. drosophilae* are projected to contract in the Eastern United States and Europe.

## 4. Discussion

Herbivorous insects, such as SWD, engage in a coevolutionary arms race with parasitoid insects whose larvae feed on the host, eventually leading to its death [97,98,99]. This property can be exploited for the biological control of invasive insects, which relies on introducing specialized parasitoids from the invader’s native range [100]. Given the economic losses caused by SWD to fruit production [16]—a consequence of its rapid expansion facilitated by globalization [101]—deploying biological control agents for SWD that share climatic niche preferences with the fly may be a promising strategy to address this global challenge [51,53,54,102]. Moreover, it is crucial to assess how these niche properties may affect the current and future potential overlap between parasitoids and hosts, since changes in these patterns can decisively influence the effectiveness of biological control in the medium and long term. As an example of climate-change–driven alterations in agricultural ecosystems, Li et al. [103] used MaxEnt to model distributions and identified increased overlap between aphids and their natural enemies, demonstrating that climate change may favor generalist enemies over specialists. These results may guide not only the choice of species to be used in biological control, but also the areas where they are likely to be most effective, both now and in the future.

In this study, we used a machine learning approach with the Random Forest algorithm to model the current and future potential distribution of parasitoids considered promising biological control agents of SWD [28] under two climate change scenarios. In doing so, we identified climatically suitable areas for these natural enemies and evaluated their potential geographic overlap with the pest. Our models reveal both similarities and differences in the potential distribution of the different SWD parasitoids, which may have implications for biological control of the pest in agricultural ecosystems under present and future climate-change contexts.

According to Langille et al. [104], temperature is among the most critical factors for population size and consequent infestation potential of SWD. Ryan et al. [105] identified mortality thresholds for this species at 5 °C (lower) and 35 °C (upper), with no adult emergence below 8.1 °C or above 30.9 °C, and optimal temperatures for development and reproduction of 28.2 °C and 22.9 °C, respectively. Abeijon et al. [96] showed that the probability of pest occurrence increases in regions where the temperature of the driest quarter ranges between 5 °C and 38 °C, and decreases in regions where the mean temperature of the wettest quarter is below 10 °C, suggesting that cold temperate climates—especially during the rainy season—may represent a significant thermal threshold for the fly’s distribution. Conversely, previous studies [106,107] identified annual precipitation and precipitation during the driest quarter as key factors influencing environmental suitability and establishment of the species. Overall, the bioclimatic variables that determine the pest’s distribution [96,106,107] largely coincide with those influencing parasitoid distributions, although precipitation-related variables appear to exert a stronger influence in the latter than temperature-related ones. When analyzed together, climatic variables such as precipitation of the wettest month (BIO13, 32.0% contribution in *L. japonica*), and coldest quarter (BIO19, 22.7% contribution in *P. vindemmiae*), along with mean diurnal range (BIO2, 24.6% contribution in *T. drosophilae*), help clarify the environmental conditions that favor or constrain the performance of these biocontrol agents.

According to our models, areas of highest climatic suitability for SWD parasitoids are predominantly located in temperate zones of the Northern Hemisphere, such as North America, Central Europe, and East Asia, indicating that these regions offer favorable conditions for the establishment of these species (Figure 3). Under future climate-change scenarios, parasitoids tend to shift toward higher latitudes, with contractions in suitability across tropical and subtropical regions (Figure 4), especially under SSP5-8.5. Under current conditions, the analysis of potential distribution revealed that *L. japonica* has the broadest climatic niche and largest area of climatic suitability, followed by *P. vindemmiae*, and *T. drosophilae*. However, distributional breadth should not be the sole factor when selecting the most effective “weapons” for biological control.

Conversely, *T. drosophilae* presents a suite of traits—such as broad geographic distribution, the ability to parasitize multiple *Drosophila* species, climatic tolerance, and occurrence in agricultural environments [40]—that increase its relevance for the biological control of *D. suzukii*. Being native to Europe [40], *T. drosophilae* is widely distributed in countries such as Italy, France, Switzerland, Austria, and the United Kingdom, where it parasitizes *D. suzukii* in vineyards, orchards, and wild areas [37,42,108,109]. In addition, according to our models, this species shows climatically suitable areas along the eastern portion of North America, Southern and Eastern Asia, and the southern regions of Africa and South America. Furthermore, our results indicate the highest likelihood spatial overlap between *T. drosophilae* and the pest at the global scale—especially in Europe but also in North America.

The parasitoid *L. japonica* is native to Asia [4] and exhibits a climatic niche more similar to that of SWD, with a preference for humid temperate regions. It is considered a promising biocontrol agent due to its adaptability and host specificity (SWD) [110]. This, combined with its potential suitability in specific regions of North America where it overlaps with SWD (Figure 4), supports its relevance for the biological control of *D. suzukii*. Indeed, several studies have reported this species in Canada and the United States in association with SWD [33,62,111]. Its broad occurrence in its native range, where it is found across different climatic zones [26,74,110], indicates substantial phenotypic plasticity, reinforcing its relevance as a biocontrol agent of *D. suzukii* on multiple continents. In this regard, our models suggest wide areas of potential overlap between *L. japonica* and *D. suzukii* in Europe (Figure 4), with a tendency to expand, particularly under future high-emission scenarios (SSP5-8.5).

*Pachycrepoideus vindemmiae* is a cosmopolitan species that has been recorded in several temperate regions of the Northern Hemisphere [112]. It stands out for its capacity to occupy varied environments, including dry and temperate climates, with strong suitability in specific regions of North America, South America, Europe, and Asia. Future projections suggest a potential expansion of overlap with SWD across much of Europe and parts of Western and Eastern Asia under both scenarios. Previous studies show that when this species encounters adequate conditions and resources for survival—namely, water and food availability—it exhibits high emergence rates. Thus, although *P. vindemmiae* shows lower specificity and control efficiency against SWD compared with *T. drosophilae* [39] or *Trichopria anastrephae* Lima, 1940 [113], its high resilience and adaptability reinforce its potential in the biological control of the fly [114].

Finally, in addition to the well-known parasitism potential of *L. japonica* in its native distribution areas [70], our models projected potential overlap between *L. japonica* and SWD in the Eastern United States and Canada, as well as across much of Europe, and this overlap is expected to persist over time. Thus, *L. japonica* may be considered by decision-makers as a regionally focused biological control option, to be deployed alongside other local or adventive parasitoids.

## 5. Conclusions

The combined use of ENM for parasitoids and potential-overlap analysis with their host is an important tool to guide decisions on the introduction and monitoring of biological control agents, enabling strategies to be adapted to changing environmental contexts. The results of this study reinforce the importance of these methodological strategies, together with information on host specificity when selecting biocontrol agents. Investments in local, ongoing field-validation studies, allied with conservation efforts and the strategic introduction of parasitoids, will be crucial to mitigate the impacts of SWD and ensure the sustainability of fruit production across different regions of the globe.

Finally, our results show how climate change can affect the potential distribution of the main natural enemies of *D. suzukii*, directly influencing the medium- and long-term efficacy of biological control strategies. The models demonstrate that parasitoids respond differently to bioclimatic variables, reflecting diverse ecological preferences and opportunities for region-specific application. Among the three species evaluated, *L. japonica*, and *T. drosophilae* stood out as more specialized parasitoids of SWD that exhibit broader areas of climatic suitability and show significant potential overlap with the pest in strategic agricultural production regions—especially in the Northern Hemisphere. For the *P. vindemmiae*, a more restricted potential distribution or the lack of specificity to SWD calls for careful planning before deploying them in management programs.

## Figures and Tables

**Table 1:** Bioclimatic variables obtained from WorldClim v. 2.1.

Bioclimatic Variable Code	Description of Each Bioclimatic Variable from WorldClim
BIO2	Mean Diurnal Range [Mean of monthly (max temp-max temp)]
BIO5	Max Temperature of Warmest Month
BIO6	Min Temperature of Coldest Month
BIO7	Temperature Annual Range (BIO5-BIO6)
BIO8	Mean Temperature of Wettest Quarter
BIO9	Mean Temperature of Driest Quarter
BIO10	Mean Temperature of Warmest Quarter
BIO11	Mean Temperature of Coldest Quarter
BIO12	Annual Precipitation
BIO13	Precipitation of Wettest Month
BIO14	Precipitation of Driest Month
BIO15	Precipitation Seasonality (Coefficient of Variation)
BIO16	Precipitation of Wettest Quarter
BIO17	Precipitation of Driest Quarter
BIO18	Precipitation of Warmest Quarter
BIO19	Precipitation of Coldest Quarter

**Table 2:** Known occurrence points, contribution of bioclimatic variables, and evaluation metrics of the distribution models of *Drosophila suzukii* parasitoid species.

Parasitoid	Known Occurrence Points	Bioclimatic Variables Contribution	AUC Mean	TSS	Threshold
*Leptopilina japonica*	41	BIO2, BIO3, BIO5, BIO8, BIO9, BIO14, BIO15, BIO18, BIO19	0.988 ± 0.020	0.518	0.264
*Pachycrepoideus vindemmiae*	63	BIO2, BIO8, BIO9, BIO10, BIO15, BIO18, BIO19	0.981 ± 0.026	0.530	0.283
*Trichopria drosophilae*	47	BIO2, BIO5, BIO7, BIO8, BIO9, BIO12, BIO13, BIO19	0.971 ± 0.048	0.550	0.245

**Table 3:** Percentage contribution (%) of bioclimatic variables to the suitability models of *L. japonica*, *P. vindemmiae*, and *T. drosophilae*.

Bioclimatic Variables	*Leptopilina japonica* (%)	*Pachycrepoideus vindemmiae* (%)	*Trichopria drosophilae* (%)
BIO2	13.2	5.4	24.6
BIO5	-	-	9.6
BIO7	-	-	6.3
BIO8	-	13.2	5.8
BIO9	-	18.1	15.6
BIO10	7.2	21.6	-
BIO11	28.5	-	-
BIO12	-	-	18.5
BIO13	32.0	-	11.1
BIO14	9.7	-	-
BIO15	2.0	10.4	-
BIO16	-	-	-
BIO17	-	-	-
BIO18	7.4	8.7	-
BIO19	-	22.7	8.7

Legend: The absence of contribution percentages for the bioclimatic variables was indicated using the symbol represented by a hyphen (-).

## Data Availability

The original contributions presented in this study are included in the article/Supplementary Material. Further inquiries can be directed to the corresponding author.

## Supplementary Materials

The following supporting information can be downloaded at: https://www.mdpi.com/article/10.3390/insects17010012/s1, Supplementary File S1: Spotted-wing drosophila parasitoid occurrence records. Supplementary File S2: Tables S1. Percentage distribution of suitable areas for Leptopilina japonica under different climate scenarios and two intervals (2021–2040 and 2041–2060), modeled using the Random Forest algorithm. Table S2. Percentage distribution of suitable areas for Pachycrepoideus vindemmiae under different climate scenarios and two intervals (2021–2040 and 2041–2060), modeled using the Random Forest algorithm. Table S3. Percentage distribution of suitable areas for Trichopria drosophilae under different climate scenarios and two intervals (2021–2040 and 2041–2060), modeled using the Random Forest algorithm.
