# Supplementary material for: Global Distribution of Three Parasitoids of Drosophila suzukii (Diptera, Drosophilidae): Present and Future Climate Change Scenarios
Source: Insects. 2025 Dec 21;17(1):12. doi: 10.3390/insects17010012 (PMC12842016; doi:10.3390/insects17010012)
Supplement: Supplementary file 1 [file insects-17-00012-s001.zip › Supplementary_file_S2.pdf]

**Table S1.** Percentage distribution of suitable areas for *Leptopilina japonica* under different climate scenarios and two intervals (2021-2040 and 2041-2060), modeled using the Random Forest algorithm.

| Countries or Territories | Baseline | Moderated scenario (SSP2-4.5) | | Pessimistic scenario (SSP5-8.5) | |
|---|---|---|---|---|---|
| | | 2021-2040 | 2041-2060 | 2021-2040 | 2041-2060 |
| Afghanistan | 3.9 | 2.2 | 2.2 | 2.4 | 1.6 |
| Albania | 0 | 4.2 | 4.2 | 2.4 | 2.4 |
| Algeria | 0 | 0.0 | 0.0 | 0.0 | 0.1 |
| Angola | 10.7 | 10.9 | 10.9 | 10.6 | 9.9 |
| Argentina | 7.2 | 3.8 | 3.8 | 4.3 | 2.2 |
| Australia | 3.6 | 0.0 | 25.7 | 0.0 | 29.35 |
| Azerbaijan | 100.0 | 100.0 | 100.0 | 100.0 | 100.0 |
| Bangladesh | 0.5 | 0.0 | 0.0 | 0.1 | 0.0 |
| Belarus | 0.9 | 15.2 | 15.2 | 8.4 | 32.4 |
| Benin | 0.0 | 0.0 | 0.0 | 0.0 | 0.0 |
| Bhutan | 0.0 | 0.0 | 0.0 | 0.0 | 0.0 |
| Bolivia | 11.5 | 3.4 | 3.4 | 3.4 | 1.6 |
| Botswana | 11.4 | 5.2 | 5.2 | 6.6 | 4.3 |
| Brazil | 68.2 | 47.8 | 47.8 | 54.0 | 42.6 |
| Bulgaria | 0.0 | 8.2 | 8.2 | 5.0 | 9.0 |
| Burkina Faso | 0.0 | 0.0 | 0.0 | 0.0 | 0.0 |
| Cameroon | 6.1 | 27.0 | 27.0 | 19.6 | 26.8 |
| Canada | 19.6 | 22.9 | 22.9 | 22.2 | 22.3 |
| Central African Republic | 16.2 | 31.4 | 31.4 | 26.0 | 33.8 |
| Chad | 3.7 | 11.7 | 11.7 | 6.2 | 9.6 |
| Chile | 0.1 | 0.1 | 0.1 | 0.0 | 0.0 |
| China | 4.2 | 1.1 | 1.1 | 1.5 | 0.8 |
| Democratic Republic of the Congo | 76.5 | 92.6 | 92.6 | 91.9 | 85.5 |
| East Timor | 0.0 | 0.0 | 0.0 | 0.0 | 0.0 |
| Egypt | 100.0 | 100.0 | 100.0 | 100.0 | 100.0 |
| Equatorial Guinea | 4.1 | 7.0 | 7.0 | 0.7 | 8.5 |
| Fiji | 9.2 | 5.7 | 7.3 | 6.2 | 6.6 |
| Finland | 0.0 | 0.1 | 0.1 | 0.0 | 0.1 |
| France | 0.0 | 0.0 | 0.0 | 0.0 | 0.0 |
| Gabon | 1.0 | 4.1 | 4.1 | 3.1 | 3.4 |
| Gambia | 20.4 | 29.0 | 29.0 | 27.8 | 30.1 |
| Georgia | 83.6 | 88.7 | 88.7 | 87.9 | 89.5 |
| Ghana | 0.0 | 0.0 | 0.0 | 0.0 | 0.0 |
| Greece | 34.3 | 45.1 | 45.1 | 39.0 | 50.3 |
| Guinea | 2.5 | 5.2 | 5.2 | 4.0 | 4.6 |
| Guinea Bissau | 28.8 | 31.6 | 31.6 | 30.6 | 32.1 |
| Honduras | 0.0 | 0.0 | 0.0 | 0.0 | 0.0 |
| Iceland | 0.2 | 11.6 | 11.6 | 8.0 | 12.7 |
| India | 0.1 | 0.0 | 0.0 | 0.0 | 0.0 |
| Indonesia | 0.0 | 0.0 | 0.0 | 0.0 | 0.0 |
| Iran | 64.6 | 61.3 | 61.3 | 62.1 | 57.6 |

| | | | | | |
|---|---|---|---|---|---|
| Iraq | 100.0 | 100.0 | 100.0 | 100.0 | 100.0 |
| Ireland | 0.0 | 0.0 | 0.0 | 0.0 | 0.0 |
| Italy | 0.0 | 0.0 | 0.0 | 0.0 | 0.0 |
| Ivory Coast | 0.0 | 0.0 | 0.0 | 0.0 | 0.0 |
| Japan | 0.0 | 0.0 | 0.0 | 0.0 | 0.0 |
| Kazakhstan | 10.6 | 20.2 | 20.2 | 18.5 | 25.2 |
| Kyrgyzstan | 0.0 | 0.0 | 0.0 | 0.0 | 0.0 |
| Laos | 33.1 | 0.3 | 0.3 | 2.1 | 2.2 |
| Lesotho | 0.0 | 0.0 | 0.0 | 0.0 | 0.0 |
| Liberia | 0.0 | 0.0 | 0.0 | 0.0 | 0.0 |
| Libya | 18.6 | 27.8 | 27.8 | 26.7 | 33.9 |
| Macedonia | 0.0 | 0.2 | 0.2 | 0.1 | 0.2 |
| Madagascar | 0.0 | 0.0 | 0.0 | 0.0 | 0.0 |
| Maldives | 0.0 | 0.0 | 0.0 | 0.0 | 0.0 |
| Mali | 0.0 | 0.0 | 0.0 | 0.0 | 0.0 |
| Malta | 0.0 | 0.0 | 0.0 | 0.0 | 0.0 |
| Mauritania | 0.1 | 0.1 | 0.1 | 0.1 | 0.2 |
| Mexico | 12.1 | 8.9 | 8.9 | 9.0 | 7.5 |
| Moldova | 0.0 | 0.0 | 0.0 | 0.0 | 0.0 |
| Mongolia | 6.3 | 3.6 | 3.6 | 4.3 | 2.9 |
| Montenegro | 0.0 | 18.4 | 18.4 | 0.0 | 21.9 |
| Morocco | 0.0 | 0.0 | 0.0 | 0.0 | 0.0 |
| Mozambique | 1.7 | 0.5 | 0.5 | 0.6 | 0.4 |
| Myanmar | 16.7 | 0.4 | 0.4 | 0.7 | 0.3 |
| Namibia | 10.4 | 2.6 | 2.6 | 3.3 | 1.7 |
| Nepal | 0.0 | 0.0 | 0.0 | 0.0 | 0.0 |
| Nicaragua | 0.0 | 0.0 | 0.0 | 0.0 | 0.0 |
| Niger | 0.0 | 0.1 | 0.1 | 0.1 | 0.1 |
| Nigeria | 5.6 | 12.0 | 12.0 | 10.4 | 14.6 |
| North Korea | 4.4 | 0.4 | 0.4 | 2.0 | 0.1 |
| Norway | 0.0 | 0.1 | 0.1 | 0.1 | 0.1 |
| Oman | 5.8 | 0.0 | 0.0 | 0.0 | 0.0 |
| Pakistan | 0.0 | 0.0 | 0.0 | 0.0 | 0.0 |
| Palau | 0.0 | 0.0 | 0.0 | 0.0 | 0.0 |
| Philippines | 0.0 | 0.0 | 0.0 | 0.0 | 0.0 |
| Portugal | 0.0 | 0.0 | 0.0 | 0.0 | 0.0 |
| Republic of Congo | 27.5 | 37.8 | 37.8 | 33.3 | 31.4 |
| Republic of Serbia | 0.1 | 6.5 | 6.5 | 1.1 | 7.2 |
| Romania | 0.0 | 38.8 | 38.8 | 28.4 | 39.3 |
| Russia | 4.6 | 7.3 | 7.3 | 7.0 | 8.5 |
| Senegal | 15.0 | 20.6 | 20.6 | 19.1 | 22.2 |
| Sierra Leone | 0.0 | 0.1 | 0.1 | 0.0 | 0.1 |
| South Africa | 17.3 | 12.5 | 12.5 | 14.0 | 11.8 |
| South Korea | 27.0 | 25.4 | 25.4 | 23.2 | 20.8 |
| Spain | 0.0 | 0.0 | 0.0 | 0.0 | 0.0 |
| Sri Lanka | 0.0 | 0.0 | 0.0 | 0.0 | 0.0 |
| Sudan | 37.5 | 52.3 | 52.3 | 46.7 | 51.9 |
| Swaziland | 0.0 | 0.0 | 0.0 | 0.0 | 0.0 |
| Sweden | 0.0 | 0.0 | 0.0 | 0.0 | 0.0 |

| | | | | | |
|---|---|---|---|---|---|
| Syria | 100.0 | 100.0 | 100.0 | 100.0 | 100.0 |
| Taiwan | 0.0 | 0.0 | 0.0 | 0.0 | 0.0 |
| Tajikistan | 0.0 | 0.0 | 0.0 | 0.0 | 0.0 |
| Thailand | 0.0 | 0.0 | 0.0 | 0.0 | 0.0 |
| Togo | 0.0 | 0.0 | 0.0 | 0.0 | 0.0 |
| Tunisia | 0.0 | 0.1 | 0.1 | 0.0 | 0.1 |
| Turkey | 72.3 | 95.7 | 95.7 | 94.0 | 98.4 |
| Turkmenistan | 74.7 | 82.3 | 82.3 | 82.2 | 80.2 |
| Ukraine | 12.9 | 18.5 | 18.5 | 19.2 | 23.7 |
| United Arab Emirates | 17.8 | 8.2 | 8.2 | 6.2 | 3.6 |
| United Kingdom | 0.0 | 0.0 | 0.0 | 0.0 | 0.0 |
| United States of America | 7.3 | 6.3 | 6.3 | 6.6 | 6.1 |
| Uzbekistan | 39.4 | 54.6 | 54.6 | 52.6 | 49.9 |
| Vietnam | 0.0 | 0.0 | 0.0 | 0.0 | 0.0 |
| Yemen | 100.0 | 100.0 | 100.0 | 100.0 | 100.0 |
| Zambia | 20.5 | 37.2 | 37.2 | 14.5 | 14.5 |
| Zimbabwe | 16.9 | 15.4 | 15.4 | 13.5 | 17.8 |
| French Polynesia | 0.0 | 0.0 | 0.0 | 0.0 | 0.0 |
| French Southern and Antarctic Lands | 56.3 | 83.9 | 83.9 | 12.6 | 100.0 |
| Greenland | | | | | |
| Guernsey | 0.0 | 0.0 | 0.0 | 0.0 | 0.0 |
| Hong Kong S.A.R. | | | | | |
| Jersey | 0.0 | 0.0 | 0.0 | 0.0 | 0.0 |
| Macao S.A.R | 0.0 | 0.0 | 0.0 | 0.0 | 0.0 |
| Saint Helena | 0.0 | 0.0 | 0.0 | 0.0 | 0.0 |
| Saint Pierre and Miquelon | 0.0 | 0.0 | 0.0 | 0.0 | 0.0 |
| Siachen Glacier | 0.0 | 0.0 | 0.0 | 0.0 | 0.0 |
| Western Sahara | 0.0 | 0.0 | 0.0 | 0.0 | 0.0 |

**Table S2.** Percentage distribution of suitable areas for *Pachycrepoideus vindemmiae* under different climate scenarios and two intervals (2021-2040 and 2041-2060), modeled using the Random Forest algorithm.

| **Countries or Territory** | **Baseline** | **Moderated scenario (SSP2-4.5)** | | **Pessimistic scenario (SSP5-8.5)** | |
|---|---|---|---|---|---|
| | | **2021-2040** | **2041-2060** | **2021-2040** | **2041-2060** |
| Afghanistan | 23.9 | 7.7 | 6.6 | 6.5 | 4.0 |
| Albania | 0.0 | 0.0 | 0.2 | 0.1 | 1.6 |
| Algeria | 0.0 | 0.0 | 0.0 | 0.0 | 0.0 |
| Angola | 0.4 | 0.1 | 0.1 | 0.0 | 0.0 |

| | | | | | |
|---|---|---|---|---|---|
| Argentina | 11.4 | 7.1 | 4.4 | 3.7 | 2.2 |
| Australia | 0.0 | 0.0 | 0.0 | 0.0 | 0.0 |
| Azerbaijan | 100.0 | 100.0 | 100.0 | 100.0 | 100.0 |
| Bangladesh | 6.6 | 2.9 | 1.7 | 2.1 | 0.5 |
| Belarus | 0.0 | 0.0 | 0.0 | 0.6 | 1.4 |
| Benin | 1.5 | 8.8 | 18.4 | 3.2 | 15.4 |
| Bhutan | 0.2 | 0.0 | 0.0 | 0.0 | 0.0 |
| Bolivia | 3.7 | 1.8 | 1.8 | 1.6 | 1.6 |
| Botswana | 10.8 | 8.1 | 7.3 | 7.1 | 5.1 |
| Brazil | 75.1 | 59.8 | 52.6 | 55.2 | 46.0 |
| Bulgaria | 0.0 | 0.0 | 0.0 | 0.0 | 4.2 |
| Burkina Faso | 0.0 | 0.0 | 0.0 | 0.0 | 0.0 |
| Cameroon | 99.3 | 99.9 | 98.6 | 99.1 | 96.9 |
| Canada | 13.8 | 14.4 | 13.2 | 12.6 | 9.0 |
| Central African Republic | 99.9 | 100.0 | 99.9 | 100.0 | 99.1 |
| Chad | 31.0 | 63.3 | 78.8 | 78.6 | 90.6 |
| Chile | 0.0 | 0.1 | 0.0 | 0.0 | 0.0 |
| China | 9.9 | 5.9 | 3.1 | 3.7 | 1.5 |
| Democratic Republic of the Congo | 0.4 | 0.4 | 0.4 | 0.4 | 0.4 |
| East Timor | 0.0 | 0.0 | 0.0 | 0.0 | 0.0 |
| Egypt | 17.5 | 1.6 | 66.7 | 8.1 | 91.6 |
| Equatorial Guinea | 100.0 | 100.0 | 100.0 | 100.0 | 100.0 |
| Fiji | 9.5 | 13.8 | 14.6 | 14.9 | 11.3 |
| Finland | 0.0 | 0.0 | 0.0 | 0.0 | 0.0 |
| France | 0.0 | 0.0 | 0.0 | 0.0 | 0.0 |
| Gabon | 99.6 | 77.8 | 60.9 | 70.1 | 47.1 |
| Gambia | 39.7 | 83.2 | 93.1 | 91.5 | 98.9 |
| Georgia | 67.2 | 78.9 | 86.0 | 84.2 | 91.4 |
| Ghana | 0.0 | 7.2 | 2.4 | 0.0 | 0.2 |
| Greece | 0.1 | 0.1 | 0.1 | 0.1 | 0.2 |
| Guinea | 36.0 | 43.7 | 45.8 | 44.9 | 45.2 |
| Guinea Bissau | 74.3 | 99.0 | 100.0 | 100.0 | 100.0 |

| | | | | | |
|---|---|---|---|---|---|
| Honduras | 0.0 | 0.0 | 0.0 | 0.0 | 0.0 |
| Iceland | 0.0 | 0.0 | 0.0 | 0.4 | 1.9 |
| India | 2.4 | 0.4 | 0.2 | 0.3 | 0.1 |
| Indonesia | 0.0 | 0.0 | 0.0 | 0.0 | 0.0 |
| Iran | 90.6 | 78.1 | 75.4 | 77.1 | 71.8 |
| Iraq | 100.0 | 100.0 | 100.0 | 100.0 | 100.0 |
| Ireland | 0.0 | 0.0 | 0.0 | 0.0 | 0.0 |
| Italy | 0.0 | 0.0 | 0.0 | 0.0 | 0.0 |
| Ivory Coast | 0.2 | 0.3 | 0.3 | 0.3 | 0.2 |
| Japan | 12.8 | 9.6 | 19.2 | 20.8 | 5.0 |
| Kazakhstan | 43.6 | 51.6 | 49.5 | 46.2 | 39.9 |
| Kyrgyzstan | 8.9 | 0.4 | 0.0 | 0.0 | 0.0 |
| Laos | 0.0 | 0.0 | 0.0 | 0.0 | 0.0 |
| Lesotho | 0.0 | 0.0 | 0.0 | 0.0 | 0.0 |
| Liberia | 2.9 | 12.4 | 15.4 | 12.4 | 16.9 |
| Libya | 2.0 | 2.6 | 4.0 | 3.3 | 4.4 |
| Macedonia | 0.0 | 0.0 | 0.0 | 0.0 | 0.0 |
| Madagascar | 0.0 | 0.0 | 0.0 | 0.0 | 0.0 |
| Maldives | 0.0 | 0.0 | 0.0 | 0.0 | 0.0 |
| Mali | 0.0 | 0.0 | 0.0 | 0.0 | 0.2 |
| Malta | 0.0 | 0.0 | 0.0 | 0.0 | 0.0 |
| Mauritania | 2.0 | 5.7 | 8.1 | 6.2 | 9.9 |
| Mexico | 20.5 | 17.1 | 15.0 | 16.2 | 13.4 |
| Moldova | 95.9 | 100.0 | 100.0 | 100.0 | 100.0 |
| Mongolia | 18.3 | 15.7 | 13.8 | 14.9 | 11.50 |
| Montenegro | 0.0 | 0.0 | 0.0 | 0.0 | 0.0 |
| Morocco | 0.0 | 0.0 | 0.1 | 0.0 | 0.9 |
| Mozambique | 3.2 | 2.1 | 1.5 | 1.5 | 1.0 |
| Myanmar | 3.1 | 0.5 | 0.4 | 0.4 | 0.3 |
| Namibia | 5.6 | 2.5 | 2.1 | 2.2 | 1.8 |
| Nepal | 0.0 | 0.0 | 0.0 | 0.0 | 0.0 |
| Nicaragua | 0.0 | 0.0 | 0.0 | 0.0 | 0.0 |
| Niger | 2.9 | 12.6 | 17.0 | 11.7 | 20.5 |

| | | | | | |
|---|---|---|---|---|---|
| Nigeria | 77.1 | 88.8 | 90.4 | 85.5 | 90.0 |
| North Korea | 1.2 | 0.0 | 0.0 | 0.0 | 0.0 |
| Norway | 0.0 | 0.0 | 0.0 | 0.0 | 0.0 |
| Oman | 100.0 | 13.2 | 10.4 | 13.2 | 10.2 |
| Pakistan | 4.1 | 0.8 | 0.3 | 0.3 | 0.1 |
| Palau | 0.0 | 0.0 | 0.0 | 0.0 | 0.0 |
| Philippines | 0.4 | 0.0 | 48.9 | 0.0 | 0.0 |
| Portugal | 0.0 | 0.0 | 0.0 | 0.0 | 0.0 |
| Republic of Congo | 62.8 | 18.6 | 7.4 | 12.6 | 4.7 |
| Republic of Serbia | 0.0 | 0.0 | 0.2 | 0.0 | 3.3 |
| Romania | 0.0 | 0.0 | 0.0 | 4.2 | 28.5 |
| Russia | 12.7 | 20.7 | 22.8 | 16.0 | 17.3 |
| Senegal | 37.8 | 63.2 | 73.9 | 68.4 | 81.5 |
| Sierra Leone | 62.0 | 80.5 | 81.6 | 77.6 | 81.0 |
| South Africa | 19.8 | 16.3 | 14.8 | 16.0 | 13.7 |
| South Korea | 44.9 | 37.4 | 34.0 | 34.5 | 23.7 |
| Spain | 0.0 | 0.0 | 0.0 | 0.0 | 0.0 |
| Sri Lanka | 1.7 | 0.2 | 0.0 | 0.0 | 0.0 |
| Sudan | 79.7 | 98.6 | 100.0 | 100.0 | 100.0 |
| Swaziland | 0.0 | 0.0 | 0.0 | 0.0 | 0.0 |
| Sweden | 0.0 | 0.0 | 0.0 | 0.0 | 0.0 |
| Syria | 100.0 | 100.0 | 100.0 | 100.0 | 100.0 |
| Taiwan | 0.0 | 0.0 | 0.0 | 0.0 | 0.0 |
| Tajikistan | 0.0 | 0.0 | 0.0 | 0.0 | 0.0 |
| Thailand | 0.0 | 0.0 | 0.0 | 0.0 | 0.0 |
| Togo | 0.0 | 0.2 | 0.6 | 0.8 | 2.0 |
| Tunisia | 0.0 | 0.0 | 0.0 | 0.0 | 0.0 |
| Turkey | 81.9 | 91.1 | 92.0 | 90.4 | 89.8 |
| Turkmenistan | 85.0 | 80.6 | 80.1 | 79.9 | 75.6 |
| Ukraine | 14.6 | 37.7 | 40.0 | 31.5 | 42.2 |
| United Arab Emirates | 100.0 | 72.8 | 47.4 | 72.8 | 30.1 |
| United Kingdom | 0.0 | 0.0 | 0.0 | 0.0 | 0.0 |

| | | | | | |
|---|---|---|---|---|---|
| United States of America | 9.4 | 7.7 | 7.2 | 7.3 | 6.4 |
| Uzbekistan | 88.9 | 85.6 | 83.9 | 83.0 | 71.3 |
| Vietnam | 0.6 | 0.1 | 0.0 | 0.0 | 0.0 |
| Yemen | 100.0 | 100.0 | 94.0 | 96.6 | 84.3 |
| Zambia | 0.0 | 0.0 | 0.0 | 0.0 | 0.0 |
| Zimbabwe | 3.6 | 0.3 | 0.1 | 0.0 | 0.0 |
| French Polynesia | 100.0 | 0.0 | 0.0 | 0.0 | 0.0 |
| French Southern and Antarctic Lands | 20.0 | 0.0 | 0.0 | 0.0 | 0.0 |
| Greenland | 28.3 | 48.4 | 53.3 | 38.9 | 41.0 |
| Guernsey | 0.0 | 0.0 | 0.0 | 0.0 | 0.0 |
| Hong Kong S.A.R. | 0.0 | 0.0 | 0.0 | 0.0 | 0.0 |
| Jersey | 0.0 | 0.0 | 0.0 | 0.0 | 0.0 |
| Macao S.A.R | 0.0 | 0.0 | 0.0 | 0.0 | 0.0 |
| Saint Helena | 0.0 | 0.0 | 0.0 | 0.0 | 0.0 |
| Saint Pierre and Miquelon | 0.0 | 0.0 | 0.0 | 0.0 | 0.0 |
| Siachen Glacier | 0.0 | 0.0 | 0.0 | 0.0 | 0.0 |
| Western Sahara | 0.0 | 0.0 | 0.6 | 0.1 | 1.9 |

**Table S3.** Percentage distribution of suitable areas for *Trichopria drosophilae* under different climate scenarios and two intervals (2021-2040 and 2041-2060), modeled using the Random Forest algorithm.

| Countries or Territory | Baseline | Moderated scenario (SSP2-4.5) | | Pessimistic scenario (SSP5-8.5) | |
|---|---|---|---|---|---|
| | | 2021-2040 | 2041-2060 | 2021-2040 | 2041-2060 |
| Afghanistan | 8.4 | 5.6 | 5.2 | 5.4 | 4.9 |
| Albania | 0.0 | 0.0 | 0.1 | 0.0 | 0.2 |
| Algeria | 0.0 | 0.0 | 0.0 | 0.0 | 0.0 |
| Angola | 63.7 | 63.3 | 62.7 | 62.5 | 59.8 |
| Argentina | 67.9 | 68.1 | 67.4 | 73.6 | 65.4 |
| Australia | 0.0 | 0.0 | 0.0 | 0.0 | 0.0 |
| Azerbaijan | 99.8 | 100.0 | 100.0 | 100.0 | 100.0 |
| Bangladesh | 0.8 | 0.2 | 0.1 | 0.2 | 0.0 |
| Belarus | 0.0 | 0.5 | 9.7 | 4.8 | 28.8 |

| | | | | | |
|---|---|---|---|---|---|
| Benin | 0.1 | 0.1 | 0.0 | 0.0 | 0.1 |
| Bhutan | 0.3 | 0.2 | 0.1 | 0.2 | 0.0 |
| Bolivia | 45.5 | 27.0 | 22.3 | 28.4 | 19.4 |
| Botswana | 47.8 | 43.3 | 40.7 | 42.2 | 37.0 |
| Brazil | 96.7 | 89.3 | 85.3 | 88.4 | 81.9 |
| Bulgaria | 0.0 | 0.6 | 0.2 | 0.0 | 0.6 |
| Burkina Faso | 0.0 | 0.0 | 0.0 | 0.0 | 0.0 |
| Cameroon | 69.1 | 79.3 | 79.5 | 78.3 | 82.9 |
| Canada | 19.1 | 18.6 | 18.1 | 18.5 | 17.3 |
| Central African Republic | 85.9 | 96.8 | 95.0 | 93.7 | 92.8 |
| Chad | 57.1 | 82.4 | 86.2 | 81.5 | 87.4 |
| Chile | 0.0 | 0.0 | 0.0 | 0.0 | 0.0 |
| China | 11.6 | 6.7 | 5.0 | 6.1 | 4.1 |
| Democratic Republic of the Congo | 36.7 | 1.7 | 1.7 | 1.7 | 1.7 |
| East Timor | 0.0 | 0.0 | 0.0 | 0.0 | 0.0 |
| Egypt | 100.0 | 100.0 | 100.0 | 100.0 | 100.0 |
| Equatorial Guinea | 24.3 | 63.7 | 66.1 | 41.3 | 33.9 |
| Fiji | 35.2 | 35.5 | 34.4 | 34.9 | 33.5 |
| Finland | 0.0 | 0.0 | 0.0 | 0.0 | 0.0 |
| France | 0.0 | 0.0 | 0.0 | 0.0 | 0.0 |
| Gabon | 90.8 | 74.0 | 73.6 | 74.3 | 48.8 |
| Gambia | 44.6 | 48.0 | 49.2 | 48.2 | 48.7 |
| Georgia | 86.1 | 90.8 | 91.1 | 91.2 | 92.2 |
| Ghana | 0.0 | 0.0 | 0.0 | 0.0 | 0.0 |
| Greece | 19.1 | 24.3 | 25.1 | 23.5 | 28.3 |
| Guinea | 46.9 | 48.9 | 48.7 | 48.2 | 47.9 |
| Guinea Bissau | 0.0 | 0.0 | 0.0 | 0.0 | 0.0 |
| Honduras | 0.0 | 0.0 | 0.0 | 0.0 | 0.0 |
| Iceland | 0.0 | 0.2 | 3.2 | 0.2 | 3.8 |
| India | 0.3 | 0.0 | 0.0 | 0.0 | 0.0 |
| Indonesia | 0.0 | 0.0 | 0.0 | 0.0 | 0.0 |
| Iran | 68.6 | 62.4 | 57.8 | 61.6 | 51.4 |

| | | | | | |
|---|---|---|---|---|---|
| Iraq | 100.0 | 100.0 | 100.0 | 100.0 | 100.0 |
| Ireland | | | | | |
| Italy | 0.0 | 0.0 | 0.0 | 0.0 | 0.0 |
| Ivory Coast | 0.0 | 0.0 | 0.0 | 0.0 | 0.0 |
| Japan | 0.0 | 0.0 | 0.0 | 0.0 | 0.0 |
| Kazakhstan | 17.6 | 26.5 | 27.0 | 25.1 | 26.9 |
| Kyrgyzstan | 1.5 | 0.8 | 0.6 | 0.7 | 0.5 |
| Laos | 100.0 | 100.0 | 100.0 | 100.0 | 100.0 |
| Lesotho | 0.0 | 0.0 | 0.0 | 0.0 | 0.0 |
| Liberia | 0.0 | 0.0 | 0.0 | 0.0 | 0.0 |
| Libya | 35.7 | 41.8 | 42.3 | 42.3 | 44.7 |
| Macedonia | 0.0 | 0.0 | 0.0 | 0.0 | 0.0 |
| Madagascar | 0.0 | 0.0 | 0.0 | 0.0 | 0.0 |
| Maldives | 0.0 | 0.0 | 0.0 | 0.0 | 0.0 |
| Mali | 0.0 | 0.0 | 0.0 | 0.0 | 0.0 |
| Malta | 0.0 | 0.0 | 0.0 | 0.0 | 0.0 |
| Mauritania | 0.7 | 1.5 | 2.0 | 1.0 | 1.9 |
| Mexico | 32.1 | 25.6 | 22.8 | 25.0 | 20.8 |
| Moldova | 0.0 | 100.0 | 100.0 | 100.0 | 100.0 |
| Mongolia | 87.0 | 25.4 | 18.8 | 20.9 | 18.1 |
| Montenegro | 0.0 | 0.0 | 0.0 | 0.0 | 0.0 |
| Morocco | 0.0 | 0.0 | 0.0 | 0.0 | 0.0 |
| Mozambique | 40.3 | 22.6 | 19.9 | 22.7 | 19.0 |
| Myanmar | 53.7 | 42.8 | 36.2 | 41.4 | 30.6 |
| Namibia | 56.5 | 52.1 | 51.6 | 51.6 | 47.9 |
| Nepal | 0.0 | 0.0 | 0.0 | 0.0 | 0.0 |
| Nicaragua | 0.0 | 0.0 | 0.0 | 0.0 | 0.0 |
| Niger | 0.1 | 9.2 | 11.1 | 5.9 | 14.9 |
| Nigeria | 20.9 | 30.1 | 31.7 | 28.2 | 34.5 |
| North Korea | 10.9 | 8.8 | 8.3 | 8.8 | 7.2 |
| Norway | 0.0 | 0.0 | 0.0 | 0.0 | 0.0 |
| Oman | 10.3 | 10.2 | 10.2 | 10.2 | 10.1 |
| Pakistan | 0.0 | 0.0 | 0.0 | 0.0 | 0.0 |

| | | | | | |
|---|---|---|---|---|---|
| Palau | 0.0 | 0.0 | 0.0 | 0.0 | 0.0 |
| Philippines | 0.0 | 0.0 | 0.0 | 0.0 | 0.0 |
| Portugal | 0.0 | 0.0 | 0.0 | 0.0 | 0.0 |
| Republic of Congo | 92.4 | 30.2 | 26.4 | 30.0 | 16.9 |
| Republic of Serbia | 0.0 | 0.0 | 1.2 | 0.0 | 1.8 |
| Romania | 0.0 | 2.5 | 2.5 | 0.0 | 2.5 |
| Russia | 5.7 | 7.2 | 7.1 | .74. | 8.5 |
| Senegal | 37.2 | 43.6 | 46.2 | 42.6 | 46.3 |
| Sierra Leone | 16.7 | 14.6 | 12.2 | 13.8 | 9.5 |
| South Africa | 26.7 | 23.2 | 21.0 | 22.9 | 19.6 |
| South Korea | 88.6 | 81.2 | 71.3 | 79.9 | 60.9 |
| Spain | 0.0 | 0.0 | 0.0 | 0.0 | 0.0 |
| Sri Lanka | 0.0 | 0.0 | 0.0 | 0.0 | 0.0 |
| Sudan | 99.8 | 100.0 | 100.0 | 100.0 | 100.0 |
| Swaziland | 0.0 | 0.0 | 0.0 | 0.0 | 0.0 |
| Sweden | 0.0 | 0.0 | 0.0 | 0.0 | 0.0 |
| Syria | 100.0 | 100.0 | 100.0 | 100.0 | 100.0 |
| Taiwan | 0.0 | 0.0 | 0.0 | 0.0 | 0.0 |
| Tajikistan | 0.6 | 0.4 | 0.3 | 0.4 | 0.3 |
| Thailand | 75.9 | 63.6 | 47.6 | 61.7 | 56.1 |
| Togo | 0.0 | 0.0 | 0.0 | 0.0 | 0.0 |
| Tunisia | 0.0 | 0.0 | 0.0 | 0.0 | 0.0 |
| Turkey | 93.2 | 95.3 | 95.2 | 95.7 | 96.7 |
| Turkmenistan | 90.5 | 85.2 | 79.3 | 82.1 | 74.7 |
| Ukraine | 10.5 | 15.5 | 20.0 | 17.3 | 25.0 |
| United Arab Emirates | 58.4 | 36.1 | 34.0 | 35.6 | 29.2 |
| United Kingdom | 0.0 | 0.0 | 0.0 | 0.0 | 0.0 |
| United States of America | 14.4 | 12.9 | 12.4 | 12.7 | 11.9 |
| Uzbekistan | 52.3 | 39.6 | 35.4 | 35.8 | 30.2 |
| Vietnam | 98.5 | 98.1 | 97.7 | 98.2 | 97.6 |
| Yemen | 100.0 | 100.0 | 100.0 | 100.0 | 100.0 |

| | | | | | |
|---|---|---|---|---|---|
| Zambia | 32.4 | 45.2 | 45.2 | 45.2 | 45.2 |
| Zimbabwe | 56.3 | 51.2 | 51.6 | 49.8 | 50.2 |
| French Polynesia | 0.0 | 0.0 | 0.0 | 0.0 | 0.0 |
| French Southern and Antarctic Lands | 49.6 | 0.0 | 0.0 | 0.0 | 0.0 |
| Greenland | 7.4 | 15.1 | 16.2 | 15.4 | 19.3 |
| Guernsey | | | | | |
| Hong Kong S.A.R. | 0.0 | 0.0 | 0.0 | 0.0 | 0.0 |
| Jersey | 0.0 | 0.0 | 0.0 | 0.0 | 0.0 |
| Macao S.A.R | 0.0 | 0.0 | 0.0 | 0.0 | 0.0 |
| Saint Helena | 0.0 | 0.0 | 0.0 | 0.0 | 0.0 |
| Saint Pierre and Miquelon | 0.0 | 0.0 | 0.0 | 0.0 | 4.5 |
| Siachen Glacier | 0.0 | 0.0 | 0.0 | 0.0 | 0.0 |
| Western Sahara | 0.0 | 0.0 | 0.0 | 0.0 | 0.0 |
